# 4-(1-Allyl-4,5-diphenyl-1*H*-imidazol-2-yl)-*N*,*N*-dimethyl­aniline

**DOI:** 10.1107/S1600536813006326

**Published:** 2013-03-09

**Authors:** Mehmet Akkurt, Frank R. Fronczek, Shaaban K. Mohamed, Avtandil H. Talybov, Adel A. E. Marzouk, Antar A. Abdelhamid

**Affiliations:** a Department of Physics, Faculty of Sciences, Erciyes University, 38039 Kayseri, Turkey; b Department of Chemistry, Louisiana State University, Baton Rouge, LA 70803-1804, USA; c Chemistry Department, Faculty of Science, Minia University, El-Minia, Egypt; d Chemistry and Environmental Division, Manchester Metropolitan University, Manchester M1 5GD, England; e Manedaliev Institute of Petrochemical Processes, National Academy of Sciences of Azerbaijan, Baku, Azerbaijan; f Pharmaceutical Chemistry Department, Faculty of Pharmacy, Al Azhar University, Egypt

## Abstract

The title compound, C_26_H_25_N_3_, crystallizes with four independent mol­ecules, 1–4, in the asymmetric unit of the triclinic unit cell. The allyl substituents on the imidazole rings adopt similar conformations in all four mol­ecules. The imadazole and the 4-and 5-substituted phenyl rings of two pairs of molecules in the asymmetric unit stack parallel to (110). In contrast, the dimethyl­aniline systems in these pairs of mol­ecules are almost normal to one another, with dihedral angles of 85.84 (10) and 85.65 (10)° between the benzene rings of the two dimethyl­aniline fragments of mol­ecules 1 and 2, and 3 and 4, respectively. The crystal structure features an extensive series of C—H⋯π inter­actions that link the mol­ecules into undulating rows along the *c* axis. The crystal studied was a pseudo-merohedral twin with twin law [-100, 0-10, 111] and the BASF parameter refined to 0.513 (3).

## Related literature

For the synthesis and bioactivity of related heterocyclic mol­ecules, see: El-Sawy *et al.* (2012 ▶); Issac *et al.* (2012 ▶); Mohamed, Abdelhamid *et al.* (2012 ▶); Soliman *et al.* (2012 ▶). For the synthesis of a similar imidazole derivative, see: Mohamed, Akkurt *et al.* (2012 ▶). For bond-length data, see: Allen *et al.* (1987 ▶).
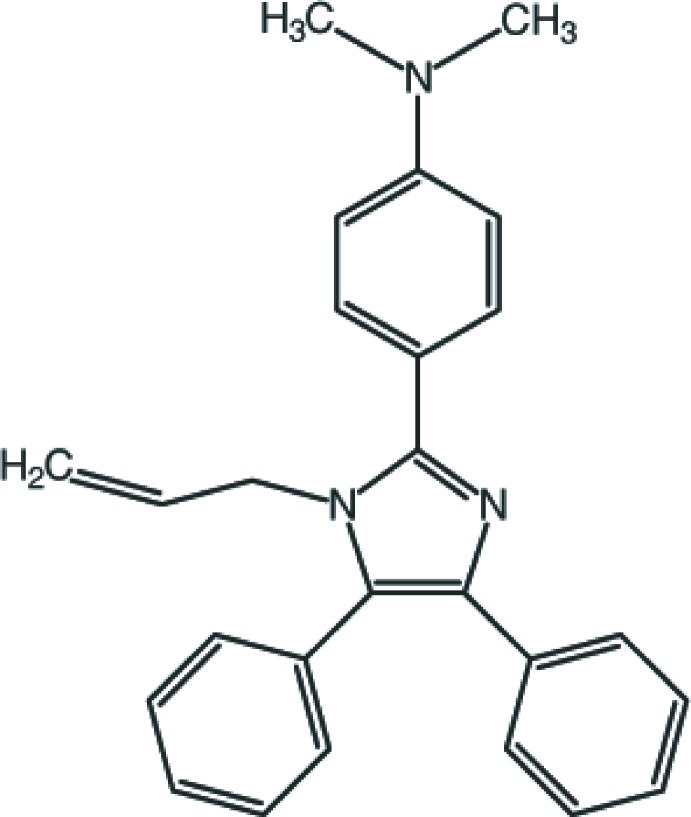


## Experimental

### 

#### Crystal data

C_26_H_25_N_3_
*M* *_r_* = 379.49
Triclinic, 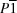

*a* = 9.4259 (7) Å
*b* = 10.2028 (7) Å
*c* = 44.721 (3) Å
α = 94.711 (2)°
β = 94.096 (2)°
γ = 107.359 (2)°
*V* = 4070.2 (5) Å^3^
*Z* = 8
Cu *K*α radiation
μ = 0.57 mm^−1^
*T* = 90 K
0.31 × 0.29 × 0.17 mm

#### Data collection

Bruker Kappa APEXII DUO diffractometer
Absorption correction: multi-scan (*SADABS*; Sheldrick, 2004 ▶) *T*_min_ = 0.844, *T*_max_ = 0.910
26145 measured reflections
13651 independent reflections
13253 reflections with *I* > 2σ(*I*)
*R*_int_ = 0.028

#### Refinement

*R*[*F* ^2^ > 2σ(*F* ^2^)] = 0.043
*wR*(*F* ^2^) = 0.118
*S* = 1.03
13651 reflections
1054 parameters
72 restraints
H-atom parameters constrained
Δρ_max_ = 0.29 e Å^−3^
Δρ_min_ = −0.29 e Å^−3^

### 

Data collection: *APEX2* (Bruker, 2005 ▶); cell refinement: *SAINT* (Bruker, 2005 ▶); data reduction: *SAINT* (Bruker, 2005 ▶); program(s) used to solve structure: *SHELXS97* (Sheldrick, 2008 ▶); program(s) used to refine structure: *SHELXL97* (Sheldrick, 2008 ▶); molecular graphics: *ORTEP-3 for Windows* (Farrugia, 2012 ▶); software used to prepare material for publication: *WinGX* (Farrugia, 2012 ▶) and *PLATON* (Spek, 2009 ▶).

## Supplementary Material

Crystal structure: contains datablock(s) global, I. DOI: 10.1107/S1600536813006326/sj5302sup1.cif

Structure factors: contains datablock(s) I. DOI: 10.1107/S1600536813006326/sj5302Isup2.hkl

Supplementary material file. DOI: 10.1107/S1600536813006326/sj5302Isup3.cml

Additional supplementary materials:  crystallographic information; 3D view; checkCIF report

## Figures and Tables

**Table 1:** Hydrogen-bond geometry (Å, °) *Cg*2, *Cg*8, *Cg*6, *Cg*4, *Cg*10, *Cg*16, *Cg*14 and *Cg*12 are the centroids of the C4–C9, C47–C52, C30–C35, C21–C26, C56–C61, C99–C104, C82–C87 and C73–C78 rings, respectively.

*D*—H⋯*A*	*D*—H	H⋯*A*	*D*⋯*A*	*D*—H⋯*A*
C10—H10*A*⋯*Cg*2^i^	0.98	2.71	3.514 (3)	139
C16—H16⋯*Cg*8	0.95	2.47	3.328 (3)	151
C37—H37*A*⋯*Cg*6^ii^	0.98	2.77	3.600 (2)	142
C42—H42⋯*Cg*4^iii^	0.95	2.49	3.345 (3)	150
C63—H63*A*⋯*Cg*10^iv^	0.98	2.73	3.595 (2)	147
C68—H68⋯*Cg*16	0.95	2.59	3.390 (3)	142
C88—H88*C*⋯*Cg*14^v^	0.98	2.74	3.509 (3)	136
C94—H94⋯*Cg*12^iii^	0.95	2.48	3.340 (3)	150

Symmetry codes: (i) 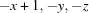; (ii) 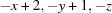; (iii) 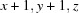; (iv) 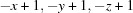; (v) 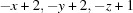.

**Fig. 1.**
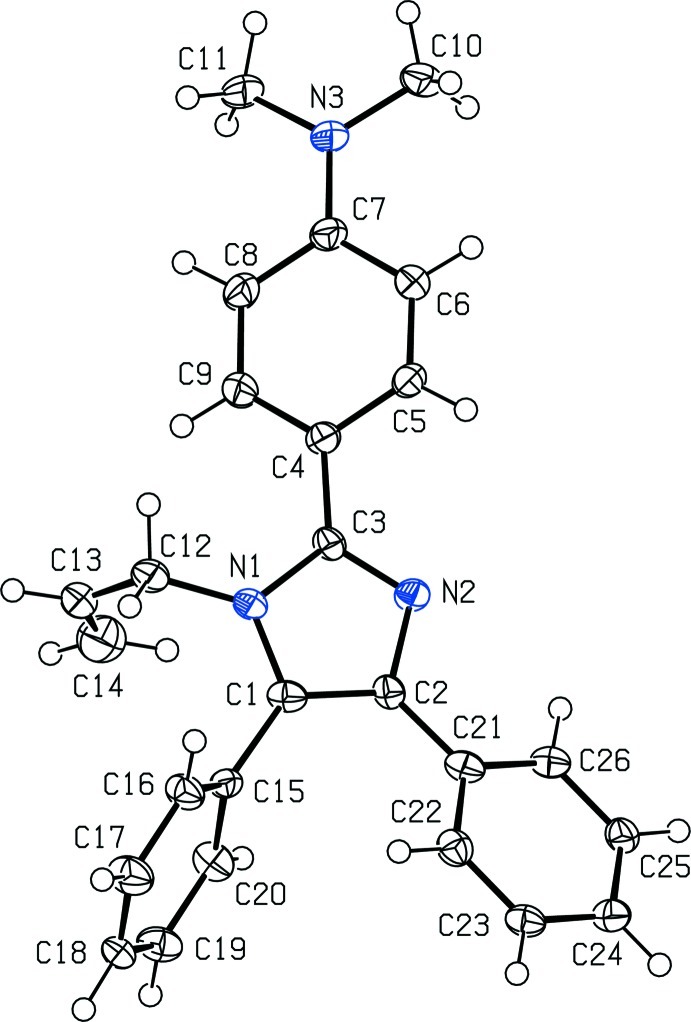
An ORTEP view of one of the four molecules in the asymmetric unit of the title compound. Displacement ellipsoids for non-H atoms are drawn at the 50% probability level.

**Fig. 2.**
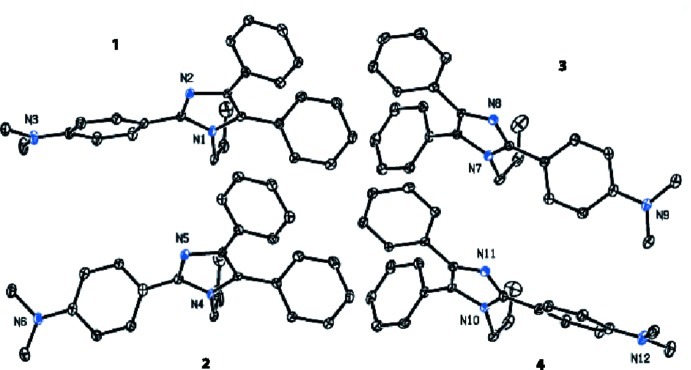
A view of the four unique molecules, 1···4, in the asymmetric unit of the title compound with the the N atoms numbered for identification purposes. Displacement ellipsoids for non-H atoms are drawn at the 30% probability level. All H atoms have been omitted for clarity.

**Fig. 3.**
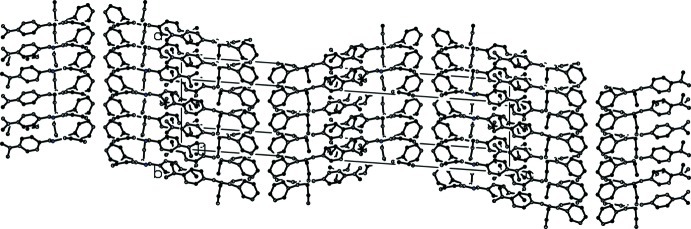
A view of the crystal packing of the title compound along the b axis. All H atoms have been omitted for clarity.

## supplementary crystallographic information

### Comment

Further to our studies on the synthesis of bioactive heterocyclic molecules such as pyrimidines and fused imidazole compounds (Soliman *et al.*, 2012), thiadiazoles (Issac *et al.* 2012), acridinones and xanthenones (Mohamed, Abdelhamid *et al.*, 2012), benzoxazinones and quinazolinones (El-Sawy *et al.*, 2012), we report herein the crystal and molecular structure of the title compound.

Figure 1 shows molecule 1 and the numbering scheme,while Figure 2 identifies the four independent molecules in the asymmetric unit. The allyl substituents on the imidazole rings adopt similar conformations in all four molecules. The –N—C(H_2_)—C(H)—C(H_2_) torsion angles of the prop-1-ene substituent on the imidazole rings are -8.5 (3), 0.4 (4), -9.6 (4) and -1.3 (4)°, respectively, for molecules 1···4. Bond lengths and angles of these four independent molecules are comparable with each other and the bond lengths are similar to values reported in the literature (Allen *et al.*, 1987).

All four of the unique molecules depart significantly from planarity. In molecule 1 (with N1), the central imidazole ring forms dihedral angles of 26.84 (12), 67.62 (13) and 27.15 (13)° with the C4–C9 benzene and C15–C20 and C21–C26 phenyl rings, respectively [the corresponding angles in molecule 2 (with N4) are 57.03 (12), 66.12 (14) and 26.74 (14)°; molecule 3 (with N7) 57.70 (11), 64.34 (13) and 27.27 (13)°, and molecule 4 (with N10) 26.85 (12), 69.03 (13) and 26.83 (12)°].

The imadazole and the 4- and 5-substituted phenyl rings of adjacent pairs of molecules stack parallel to (110). In contrast, the dimethylaniline systems in these pairs of molecules are almost normal to one another with dihedral angles of 85.84 (10)° between the C5···C9 and C30···C35 benzene rings of molecules 1 and 2 and 85.65 (10)° between the C56···C61 and C82···C87 benzene rings of molecules 3 and 4.

The crystal structure is stabilized by an extensive series of C—H···π interactions (Table 1), that link the molecules into undulating rows along the *c* axis. Figure 3 shows the crystal packing of the title compound along the *b* axis.

### Experimental

The title compound was prepared among series of imidazole derivatives according to our reported method (Mohamed, Akkurt *et al.*, 2012). Suitable single crystals of (I) were obtained by the slow evaporation method using ethanol as a solvent. *M*.p. 403 – 405 K.

### Refinement

All H atoms were located geometrically and refined using a riding model with d(C—H) = 0.95–0.99 Å and, *U*_iso_(methyl H) = 1.5 *U*_eq_(C) and *U*_iso_(H) = 1.2 *U*_eq_(C) for other H atoms. The C12, C13, C14, C38, C39, C40, C64, C65, C66, C90, C91 and C92 atoms were refined anisotropically with the *U^ij^* values restrained to behave isotropically, with the ISOR instruction. The (0 - 1 10 and -1 - 1 1) reflections were probably affected by the beamstop and were omitted from the refinement. The structure was refined as a pseudo-merohedral twin with twin law [-1 0 0, 0 - 1 0, 1 1 1]. The final refined BASF value is 0.513 (3).

### Crystal data

**Table:**

C_26_H_25_N_3_	*Z* = 8
*M**_r_* = 379.49	*F*(000) = 1616
Triclinic, *P*1	*D*_x_ = 1.239 Mg m^−^^3^
Hall symbol: -P 1	Cu *K*α radiation, λ = 1.54184 Å
*a* = 9.4259 (7) Å	Cell parameters from 9806 reflections
*b* = 10.2028 (7) Å	θ = 4.6–67.5°
*c* = 44.721 (3) Å	µ = 0.57 mm^−^^1^
α = 94.711 (2)°	*T* = 90 K
β = 94.096 (2)°	Fragment, colourless
γ = 107.359 (2)°	0.31 × 0.29 × 0.17 mm
*V* = 4070.2 (5) Å^3^	

### Data collection

**Table:**

Bruker Kappa APEXII DUO diffractometer	13651 independent reflections
Radiation source: IµS microfocus	13253 reflections with *I* > 2σ(*I*)
QUAZAR multilayer optics monochromator	*R*_int_ = 0.028
φ and ω scans	θ_max_ = 68.1°, θ_min_ = 4.6°
Absorption correction: multi-scan (*SADABS*; Sheldrick, 2004)	*h* = −10→11
*T*_min_ = 0.844, *T*_max_ = 0.910	*k* = −6→12
26145 measured reflections	*l* = −53→53

### Refinement

**Table:**

Refinement on *F*^2^	Primary atom site location: structure-invariant direct methods
Least-squares matrix: full	Secondary atom site location: difference Fourier map
*R*[*F*^2^ > 2σ(*F*^2^)] = 0.043	Hydrogen site location: inferred from neighbouring sites
*wR*(*F*^2^) = 0.118	H-atom parameters constrained
*S* = 1.03	*w* = 1/[σ^2^(*F*_o_^2^) + (0.0564*P*)^2^ + 1.1958*P*] where *P* = (*F*_o_^2^ + 2*F*_c_^2^)/3
13651 reflections	(Δ/σ)_max_ = 0.001
1054 parameters	Δρ_max_ = 0.29 e Å^−^^3^
72 restraints	Δρ_min_ = −0.29 e Å^−^^3^

### Special details

**Table:**

Geometry. Bond distances, angles *etc*. have been calculated using the rounded fractional coordinates. All su's are estimated from the variances of the (full) variance-covariance matrix. The cell e.s.d.'s are taken into account in the estimation of distances, angles and torsion angles
Refinement. Refinement on *F*^2^ for ALL reflections except those flagged by the user for potential systematic errors. Weighted *R*-factors *wR* and all goodnesses of fit *S* are based on *F*^2^, conventional *R*-factors *R* are based on *F*, with *F* set to zero for negative *F*^2^. The observed criterion of *F*^2^ > σ(*F*^2^) is used only for calculating -*R*-factor-obs *etc*. and is not relevant to the choice of reflections for refinement. *R*-factors based on *F*^2^ are statistically about twice as large as those based on *F*, and *R*-factors based on ALL data will be even larger.

### Fractional atomic coordinates and isotropic or equivalent isotropic displacement parameters (Å^2^)

**Table:**

	*x*	*y*	*z*	*U*_iso_*/*U*_eq_	
N1	0.2373 (2)	0.0763 (2)	0.11871 (4)	0.0225 (5)	
N2	0.2148 (2)	−0.1476 (2)	0.10855 (4)	0.0214 (5)	
N3	0.2954 (2)	−0.0143 (2)	−0.02847 (4)	0.0318 (6)	
C1	0.2128 (2)	0.0195 (3)	0.14483 (5)	0.0218 (6)	
C2	0.2006 (2)	−0.1205 (2)	0.13908 (5)	0.0188 (6)	
C3	0.2368 (2)	−0.0300 (2)	0.09676 (5)	0.0212 (6)	
C4	0.2551 (2)	−0.0165 (2)	0.06473 (5)	0.0229 (6)	
C5	0.3112 (2)	−0.1115 (2)	0.04883 (4)	0.0255 (6)	
C6	0.3242 (2)	−0.1122 (2)	0.01854 (5)	0.0275 (6)	
C7	0.2839 (2)	−0.0157 (2)	0.00186 (5)	0.0272 (6)	
C8	0.2299 (3)	0.0816 (2)	0.01796 (5)	0.0305 (7)	
C9	0.2160 (3)	0.0794 (2)	0.04828 (5)	0.0294 (7)	
C10	0.3390 (3)	−0.1217 (2)	−0.04546 (5)	0.0338 (7)	
C11	0.2454 (4)	0.0836 (3)	−0.04489 (5)	0.0426 (9)	
C12	0.2655 (3)	0.2194 (3)	0.11544 (5)	0.0293 (7)	
C13	0.1396 (3)	0.2691 (3)	0.11371 (5)	0.0326 (7)	
C14	−0.0106 (3)	0.1854 (4)	0.11112 (7)	0.0455 (10)	
C15	0.1976 (3)	0.0965 (3)	0.17334 (5)	0.0204 (7)	
C16	0.3228 (3)	0.1963 (3)	0.18926 (6)	0.0266 (7)	
C17	0.3100 (3)	0.2654 (3)	0.21664 (6)	0.0342 (8)	
C18	0.1747 (3)	0.2333 (3)	0.22898 (5)	0.0271 (7)	
C19	0.0506 (3)	0.1338 (3)	0.21314 (6)	0.0301 (7)	
C20	0.0635 (3)	0.0657 (3)	0.18609 (6)	0.0271 (7)	
C21	0.1826 (2)	−0.2274 (3)	0.15890 (5)	0.0235 (7)	
C22	0.2333 (3)	−0.1946 (3)	0.19014 (6)	0.0245 (7)	
C23	0.2137 (2)	−0.2950 (3)	0.20945 (5)	0.0241 (6)	
C24	0.1430 (3)	−0.4351 (3)	0.19829 (6)	0.0289 (7)	
C25	0.0963 (3)	−0.4674 (3)	0.16783 (6)	0.0247 (6)	
C26	0.1177 (3)	−0.3648 (3)	0.14864 (5)	0.0242 (6)	
N4	0.7512 (2)	0.6001 (2)	0.12200 (4)	0.0209 (5)	
N5	0.7198 (2)	0.3771 (2)	0.10856 (4)	0.0233 (6)	
N6	0.8391 (2)	0.60149 (19)	−0.02239 (4)	0.0297 (6)	
C27	0.7197 (2)	0.5349 (3)	0.14697 (5)	0.0217 (6)	
C28	0.7030 (2)	0.3949 (3)	0.13924 (5)	0.0214 (7)	
C29	0.7482 (3)	0.4988 (3)	0.09882 (5)	0.0242 (7)	
C30	0.7712 (3)	0.5301 (2)	0.06782 (5)	0.0253 (7)	
C31	0.6640 (2)	0.4604 (2)	0.04421 (4)	0.0236 (6)	
C32	0.6854 (2)	0.4832 (2)	0.01452 (4)	0.0245 (6)	
C33	0.8168 (2)	0.5775 (2)	0.00714 (4)	0.0242 (6)	
C34	0.9247 (2)	0.6485 (2)	0.03105 (4)	0.0256 (6)	
C35	0.9023 (2)	0.6255 (2)	0.06057 (4)	0.0246 (6)	
C36	0.7416 (3)	0.5088 (3)	−0.04678 (5)	0.0340 (7)	
C37	0.9868 (2)	0.6789 (2)	−0.02920 (5)	0.0319 (7)	
C38	0.7531 (3)	0.7391 (3)	0.11852 (5)	0.0330 (7)	
C39	0.6072 (5)	0.7582 (3)	0.11284 (7)	0.0582 (13)	
C40	0.4714 (3)	0.6564 (3)	0.11080 (6)	0.0381 (8)	
C41	0.7057 (3)	0.6071 (3)	0.17633 (5)	0.0215 (7)	
C42	0.8321 (3)	0.7035 (3)	0.19241 (5)	0.0240 (6)	
C43	0.8207 (3)	0.7712 (3)	0.22014 (5)	0.0296 (7)	
C44	0.6851 (3)	0.7409 (3)	0.23226 (5)	0.0274 (7)	
C45	0.5594 (3)	0.6443 (3)	0.21616 (6)	0.0319 (8)	
C46	0.5707 (3)	0.5774 (3)	0.18895 (6)	0.0287 (7)	
C47	0.6799 (3)	0.2826 (3)	0.15742 (5)	0.0244 (7)	
C48	0.7280 (3)	0.3056 (3)	0.18890 (6)	0.0272 (7)	
C49	0.7047 (3)	0.2001 (3)	0.20674 (6)	0.0301 (8)	
C50	0.6320 (3)	0.0621 (3)	0.19375 (6)	0.0318 (8)	
C51	0.5880 (3)	0.0386 (3)	0.16323 (6)	0.0294 (7)	
C52	0.6133 (3)	0.1470 (3)	0.14535 (5)	0.0269 (7)	
N7	0.1237 (2)	0.4775 (2)	0.37921 (4)	0.0237 (6)	
N8	0.1092 (2)	0.2697 (2)	0.39208 (4)	0.0223 (5)	
N9	0.3624 (2)	0.6246 (2)	0.52295 (4)	0.0296 (6)	
C53	0.0650 (2)	0.3835 (3)	0.35288 (5)	0.0215 (6)	
C54	0.0609 (2)	0.2603 (3)	0.36195 (5)	0.0210 (6)	
C55	0.1460 (2)	0.4029 (3)	0.40159 (5)	0.0202 (6)	
C56	0.2035 (2)	0.4637 (2)	0.43285 (5)	0.0215 (6)	
C57	0.1198 (2)	0.4171 (2)	0.45651 (4)	0.0249 (6)	
C58	0.1709 (2)	0.4695 (2)	0.48622 (4)	0.0250 (6)	
C59	0.3097 (2)	0.5707 (2)	0.49347 (4)	0.0245 (6)	
C60	0.3935 (2)	0.6186 (2)	0.46961 (4)	0.0261 (6)	
C61	0.3402 (2)	0.5655 (2)	0.43999 (4)	0.0249 (6)	
C62	0.2913 (3)	0.5566 (3)	0.54715 (5)	0.0357 (8)	
C63	0.5181 (2)	0.7089 (2)	0.52963 (5)	0.0318 (6)	
C64	0.1307 (3)	0.6229 (3)	0.38245 (5)	0.0285 (7)	
C65	−0.0166 (3)	0.6434 (3)	0.38863 (6)	0.0370 (8)	
C66	−0.1441 (4)	0.5484 (4)	0.38728 (8)	0.0576 (11)	
C67	0.0223 (3)	0.4310 (3)	0.32416 (5)	0.0217 (6)	
C68	0.1276 (3)	0.5165 (3)	0.30879 (6)	0.0273 (7)	
C69	0.0867 (3)	0.5522 (3)	0.28131 (6)	0.0305 (7)	
C70	−0.0601 (3)	0.5072 (3)	0.26886 (6)	0.0306 (8)	
C71	−0.1656 (3)	0.4231 (3)	0.28393 (5)	0.0311 (8)	
C72	−0.1276 (3)	0.3864 (3)	0.31209 (6)	0.0291 (7)	
C73	0.0208 (2)	0.1250 (3)	0.34245 (5)	0.0218 (6)	
C74	0.0372 (2)	0.1210 (3)	0.31201 (5)	0.0232 (7)	
C75	−0.0043 (3)	−0.0062 (3)	0.29449 (5)	0.0295 (7)	
C76	−0.0585 (3)	−0.1265 (3)	0.30642 (6)	0.0312 (8)	
C77	−0.0748 (3)	−0.1243 (3)	0.33720 (6)	0.0319 (7)	
C78	−0.0350 (2)	0.0003 (3)	0.35543 (5)	0.0248 (7)	
N10	0.6152 (2)	0.9552 (2)	0.38150 (4)	0.0214 (5)	
N11	0.6021 (2)	0.7439 (2)	0.39089 (4)	0.0229 (5)	
N12	0.8247 (2)	1.0142 (2)	0.52795 (4)	0.0344 (6)	
C79	0.5611 (2)	0.8690 (2)	0.35378 (5)	0.0201 (6)	
C80	0.5576 (2)	0.7430 (3)	0.36100 (5)	0.0229 (7)	
C81	0.6361 (2)	0.8744 (3)	0.40265 (5)	0.0227 (6)	
C82	0.6905 (2)	0.9192 (3)	0.43469 (5)	0.0255 (7)	
C83	0.7593 (2)	0.8385 (2)	0.45046 (4)	0.0268 (6)	
C84	0.8034 (2)	0.8686 (2)	0.48108 (5)	0.0282 (6)	
C85	0.7808 (2)	0.9826 (2)	0.49751 (5)	0.0276 (7)	
C86	0.7106 (3)	1.0622 (3)	0.48165 (5)	0.0299 (7)	
C87	0.6651 (2)	1.0298 (2)	0.45109 (4)	0.0288 (7)	
C88	0.8852 (3)	0.9236 (3)	0.54479 (5)	0.0352 (7)	
C89	0.7915 (3)	1.1269 (3)	0.54418 (5)	0.0420 (9)	
C90	0.6479 (3)	1.1060 (3)	0.38405 (5)	0.0273 (7)	
C91	0.5167 (3)	1.1531 (3)	0.38677 (6)	0.0328 (8)	
C92	0.3790 (4)	1.0781 (3)	0.38664 (8)	0.0510 (10)	
C93	0.5231 (3)	0.9231 (3)	0.32560 (5)	0.0213 (7)	
C94	0.6311 (3)	1.0059 (3)	0.31025 (6)	0.0298 (7)	
C95	0.5938 (3)	1.0464 (3)	0.28294 (6)	0.0303 (8)	
C96	0.4471 (3)	1.0081 (3)	0.27067 (5)	0.0292 (7)	
C97	0.3384 (3)	0.9272 (3)	0.28552 (6)	0.0318 (8)	
C98	0.3734 (3)	0.8861 (3)	0.31346 (6)	0.0296 (7)	
C99	0.5169 (2)	0.6121 (2)	0.34015 (5)	0.0188 (6)	
C100	0.5338 (2)	0.6144 (3)	0.30981 (5)	0.0223 (7)	
C101	0.4907 (3)	0.4902 (3)	0.29093 (5)	0.0281 (7)	
C102	0.4342 (3)	0.3672 (3)	0.30169 (5)	0.0259 (7)	
C103	0.4192 (3)	0.3623 (3)	0.33246 (6)	0.0267 (7)	
C104	0.4601 (2)	0.4842 (3)	0.35182 (5)	0.0220 (6)	
H5	0.34100	−0.17740	0.05940	0.0310*	
H6	0.36130	−0.17930	0.00860	0.0330*	
H8	0.20280	0.14970	0.00760	0.0370*	
H9	0.17830	0.14570	0.05840	0.0350*	
H10A	0.43510	−0.12560	−0.03630	0.0510*	
H10B	0.34860	−0.10060	−0.06640	0.0510*	
H10C	0.26280	−0.21110	−0.04520	0.0510*	
H11A	0.13810	0.06660	−0.04370	0.0640*	
H11B	0.26450	0.07240	−0.06610	0.0640*	
H11C	0.29980	0.17790	−0.03600	0.0640*	
H12A	0.33610	0.27320	0.13270	0.0350*	
H12B	0.31630	0.23900	0.09690	0.0350*	
H13	0.15820	0.36620	0.11440	0.0390*	
H14A	−0.03380	0.08790	0.11040	0.0550*	
H14B	−0.08870	0.22650	0.11010	0.0550*	
H16	0.41670	0.21670	0.18130	0.0320*	
H17	0.39440	0.33470	0.22690	0.0410*	
H18	0.16640	0.27830	0.24790	0.0330*	
H19	−0.04340	0.11310	0.22110	0.0360*	
H20	−0.02120	−0.00370	0.17600	0.0320*	
H22	0.28180	−0.10100	0.19780	0.0290*	
H23	0.24750	−0.27050	0.23030	0.0290*	
H24	0.12810	−0.50550	0.21140	0.0350*	
H25	0.04910	−0.56100	0.16000	0.0300*	
H26	0.08650	−0.39010	0.12770	0.0290*	
H31	0.57410	0.39570	0.04850	0.0280*	
H32	0.61000	0.43420	−0.00110	0.0290*	
H34	1.01460	0.71340	0.02680	0.0310*	
H35	0.97680	0.67520	0.07630	0.0290*	
H36A	0.63900	0.51130	−0.04550	0.0510*	
H36B	0.74500	0.41450	−0.04520	0.0510*	
H36C	0.77470	0.53780	−0.06610	0.0510*	
H37A	1.06000	0.63620	−0.02130	0.0480*	
H37B	1.01210	0.77430	−0.01980	0.0480*	
H37C	0.98820	0.67850	−0.05110	0.0480*	
H38A	0.81220	0.77090	0.10160	0.0400*	
H38B	0.80520	0.79890	0.13700	0.0400*	
H39	0.60530	0.84920	0.11030	0.0700*	
H40A	0.46780	0.56380	0.11320	0.0460*	
H40B	0.38140	0.67890	0.10700	0.0460*	
H42	0.92590	0.72280	0.18440	0.0290*	
H43	0.90620	0.83810	0.23070	0.0350*	
H44	0.67740	0.78530	0.25130	0.0330*	
H45	0.46540	0.62480	0.22410	0.0380*	
H46	0.48510	0.50990	0.17860	0.0340*	
H48	0.77780	0.39730	0.19780	0.0330*	
H49	0.73700	0.21880	0.22770	0.0360*	
H50	0.61410	−0.01230	0.20590	0.0380*	
H51	0.53970	−0.05320	0.15430	0.0350*	
H52	0.58390	0.12730	0.12430	0.0320*	
H57	0.02570	0.34800	0.45220	0.0300*	
H58	0.11100	0.43640	0.50180	0.0300*	
H60	0.48760	0.68790	0.47380	0.0310*	
H61	0.39850	0.59960	0.42420	0.0300*	
H62A	0.18290	0.53700	0.54340	0.0540*	
H62B	0.32760	0.61640	0.56620	0.0540*	
H62C	0.31470	0.46970	0.54850	0.0540*	
H63A	0.58310	0.65580	0.52280	0.0480*	
H63B	0.54040	0.73530	0.55140	0.0480*	
H63C	0.53560	0.79220	0.51920	0.0480*	
H64A	0.16220	0.66350	0.36370	0.0340*	
H64B	0.20720	0.67270	0.39920	0.0340*	
H65	−0.01580	0.73570	0.39410	0.0440*	
H66A	−0.15090	0.45450	0.38190	0.0690*	
H66B	−0.23090	0.57250	0.39160	0.0690*	
H68	0.22880	0.55090	0.31720	0.0330*	
H69	0.16080	0.60900	0.27060	0.0370*	
H70	−0.08730	0.53440	0.25000	0.0370*	
H71	−0.26630	0.38910	0.27510	0.0370*	
H72	−0.20240	0.33180	0.32290	0.0350*	
H74	0.07640	0.20410	0.30310	0.0280*	
H75	0.00570	−0.00840	0.27350	0.0350*	
H76	−0.08520	−0.21180	0.29400	0.0370*	
H77	−0.11330	−0.20850	0.34570	0.0380*	
H78	−0.04510	0.00180	0.37640	0.0300*	
H83	0.77620	0.76070	0.43990	0.0320*	
H84	0.84970	0.81120	0.49110	0.0340*	
H86	0.69360	1.14030	0.49210	0.0360*	
H87	0.61530	1.08480	0.44110	0.0350*	
H88A	0.80750	0.83520	0.54510	0.0530*	
H88B	0.91940	0.96690	0.56550	0.0530*	
H88C	0.96950	0.90780	0.53510	0.0530*	
H89A	0.84930	1.21410	0.53740	0.0630*	
H89B	0.81790	1.12790	0.56580	0.0630*	
H89C	0.68460	1.11560	0.54050	0.0630*	
H90A	0.72070	1.14710	0.40190	0.0330*	
H90B	0.69560	1.14080	0.36600	0.0330*	
H91	0.53470	1.25020	0.38890	0.0390*	
H92A	0.35400	0.98020	0.38460	0.0610*	
H92B	0.30290	1.12090	0.38860	0.0610*	
H94	0.73240	1.03530	0.31860	0.0360*	
H95	0.67000	1.10140	0.27240	0.0360*	
H96	0.42190	1.03790	0.25200	0.0350*	
H97	0.23770	0.89830	0.27680	0.0380*	
H98	0.29640	0.83350	0.32410	0.0360*	
H100	0.57430	0.69960	0.30180	0.0270*	
H101	0.50130	0.49230	0.27000	0.0340*	
H102	0.40480	0.28430	0.28840	0.0310*	
H103	0.38120	0.27590	0.34010	0.0320*	
H104	0.44990	0.48150	0.37280	0.0260*	

### Atomic displacement parameters (Å^2^)

**Table:**

	*U*^11^	*U*^22^	*U*^33^	*U*^12^	*U*^13^	*U*^23^
N1	0.0297 (9)	0.0220 (10)	0.0152 (9)	0.0078 (8)	0.0023 (7)	−0.0011 (7)
N2	0.0254 (9)	0.0224 (10)	0.0146 (9)	0.0060 (7)	−0.0025 (7)	0.0006 (7)
N3	0.0525 (12)	0.0296 (11)	0.0173 (9)	0.0165 (9)	0.0086 (8)	0.0059 (7)
C1	0.0192 (10)	0.0244 (12)	0.0164 (10)	−0.0001 (8)	−0.0005 (8)	−0.0015 (9)
C2	0.0192 (9)	0.0185 (11)	0.0163 (10)	0.0047 (8)	−0.0038 (8)	−0.0028 (8)
C3	0.0246 (10)	0.0189 (11)	0.0174 (10)	0.0045 (8)	0.0000 (8)	−0.0030 (8)
C4	0.0302 (11)	0.0175 (11)	0.0165 (10)	0.0015 (9)	0.0015 (8)	−0.0008 (8)
C5	0.0342 (11)	0.0202 (11)	0.0212 (10)	0.0064 (9)	0.0030 (8)	0.0046 (8)
C6	0.0360 (11)	0.0237 (11)	0.0239 (10)	0.0101 (9)	0.0067 (8)	0.0016 (8)
C7	0.0378 (12)	0.0229 (11)	0.0191 (10)	0.0063 (9)	0.0039 (8)	0.0027 (8)
C8	0.0453 (13)	0.0260 (12)	0.0221 (10)	0.0133 (10)	0.0022 (9)	0.0056 (9)
C9	0.0426 (12)	0.0274 (12)	0.0210 (10)	0.0150 (10)	0.0043 (8)	0.0017 (8)
C10	0.0466 (13)	0.0324 (13)	0.0215 (10)	0.0104 (10)	0.0086 (9)	−0.0002 (9)
C11	0.0782 (19)	0.0390 (15)	0.0173 (11)	0.0249 (14)	0.0102 (11)	0.0102 (10)
C12	0.0465 (13)	0.0252 (13)	0.0139 (10)	0.0094 (10)	0.0014 (9)	−0.0030 (9)
C13	0.0487 (14)	0.0265 (12)	0.0244 (11)	0.0132 (11)	0.0101 (10)	0.0018 (10)
C14	0.0493 (16)	0.0563 (19)	0.0388 (14)	0.0272 (14)	0.0028 (12)	0.0098 (13)
C15	0.0245 (11)	0.0201 (12)	0.0164 (11)	0.0059 (9)	0.0028 (8)	0.0035 (9)
C16	0.0280 (11)	0.0229 (12)	0.0243 (11)	0.0021 (10)	0.0049 (10)	−0.0042 (10)
C17	0.0342 (13)	0.0316 (14)	0.0292 (13)	0.0003 (11)	0.0058 (10)	−0.0055 (11)
C18	0.0419 (13)	0.0228 (13)	0.0178 (11)	0.0118 (10)	0.0068 (10)	−0.0019 (9)
C19	0.0240 (11)	0.0404 (15)	0.0273 (12)	0.0113 (10)	0.0067 (9)	0.0032 (11)
C20	0.0214 (11)	0.0301 (14)	0.0265 (12)	0.0057 (10)	−0.0014 (9)	−0.0033 (10)
C21	0.0196 (10)	0.0296 (13)	0.0200 (11)	0.0060 (9)	0.0044 (9)	−0.0001 (10)
C22	0.0209 (10)	0.0261 (13)	0.0268 (12)	0.0081 (9)	0.0030 (9)	0.0004 (10)
C23	0.0242 (10)	0.0283 (13)	0.0171 (10)	0.0053 (9)	0.0006 (8)	−0.0017 (9)
C24	0.0339 (12)	0.0322 (14)	0.0257 (12)	0.0143 (10)	0.0113 (9)	0.0086 (10)
C25	0.0289 (11)	0.0193 (11)	0.0238 (11)	0.0047 (9)	0.0048 (9)	−0.0019 (9)
C26	0.0244 (10)	0.0292 (13)	0.0174 (10)	0.0064 (9)	0.0024 (8)	0.0008 (9)
N4	0.0239 (9)	0.0236 (10)	0.0115 (8)	0.0040 (7)	0.0007 (6)	−0.0067 (7)
N5	0.0238 (9)	0.0281 (11)	0.0154 (9)	0.0058 (8)	−0.0032 (7)	−0.0005 (8)
N6	0.0399 (10)	0.0293 (11)	0.0184 (9)	0.0081 (8)	0.0044 (7)	0.0029 (7)
C27	0.0176 (9)	0.0212 (11)	0.0190 (11)	−0.0035 (8)	−0.0008 (8)	−0.0006 (9)
C28	0.0188 (10)	0.0263 (13)	0.0165 (11)	0.0058 (9)	−0.0028 (8)	−0.0036 (9)
C29	0.0234 (10)	0.0271 (13)	0.0195 (11)	0.0053 (9)	0.0018 (8)	−0.0025 (9)
C30	0.0314 (11)	0.0295 (13)	0.0185 (10)	0.0148 (10)	0.0031 (8)	0.0017 (9)
C31	0.0237 (10)	0.0252 (12)	0.0210 (10)	0.0067 (8)	0.0026 (7)	−0.0001 (8)
C32	0.0288 (10)	0.0257 (12)	0.0200 (10)	0.0113 (9)	−0.0012 (8)	−0.0005 (8)
C33	0.0352 (11)	0.0220 (11)	0.0184 (10)	0.0137 (9)	0.0013 (8)	0.0019 (8)
C34	0.0303 (10)	0.0244 (11)	0.0221 (10)	0.0076 (9)	0.0042 (8)	0.0035 (8)
C35	0.0279 (10)	0.0242 (11)	0.0201 (10)	0.0068 (9)	0.0002 (8)	0.0003 (8)
C36	0.0475 (13)	0.0376 (14)	0.0164 (10)	0.0133 (11)	0.0016 (9)	−0.0003 (10)
C37	0.0407 (12)	0.0378 (13)	0.0208 (10)	0.0153 (10)	0.0079 (8)	0.0077 (9)
C38	0.0486 (14)	0.0254 (13)	0.0159 (11)	−0.0018 (11)	0.0066 (10)	−0.0031 (9)
C39	0.120 (3)	0.0402 (18)	0.0378 (15)	0.048 (2)	0.0445 (18)	0.0177 (14)
C40	0.0389 (13)	0.0507 (16)	0.0309 (12)	0.0208 (12)	0.0067 (10)	0.0096 (11)
C41	0.0218 (10)	0.0236 (13)	0.0187 (11)	0.0055 (9)	0.0041 (8)	0.0037 (9)
C42	0.0229 (10)	0.0252 (12)	0.0211 (11)	0.0047 (9)	0.0015 (9)	−0.0019 (9)
C43	0.0294 (12)	0.0312 (14)	0.0217 (11)	0.0021 (10)	0.0015 (9)	−0.0053 (10)
C44	0.0421 (13)	0.0274 (14)	0.0151 (10)	0.0142 (11)	0.0055 (9)	0.0000 (9)
C45	0.0241 (11)	0.0461 (17)	0.0294 (13)	0.0135 (11)	0.0087 (10)	0.0101 (12)
C46	0.0202 (11)	0.0377 (15)	0.0248 (11)	0.0050 (10)	−0.0008 (9)	0.0021 (11)
C47	0.0211 (10)	0.0322 (14)	0.0190 (11)	0.0064 (9)	0.0046 (9)	0.0020 (10)
C48	0.0234 (11)	0.0278 (14)	0.0291 (13)	0.0070 (10)	0.0019 (10)	0.0001 (11)
C49	0.0304 (12)	0.0344 (15)	0.0233 (12)	0.0069 (10)	0.0035 (10)	0.0019 (10)
C50	0.0359 (13)	0.0326 (14)	0.0288 (12)	0.0111 (11)	0.0103 (10)	0.0066 (11)
C51	0.0321 (11)	0.0207 (12)	0.0321 (13)	0.0036 (10)	0.0076 (10)	−0.0020 (10)
C52	0.0279 (11)	0.0295 (13)	0.0212 (11)	0.0059 (10)	0.0044 (9)	−0.0002 (10)
N7	0.0221 (9)	0.0240 (11)	0.0218 (9)	0.0046 (7)	0.0005 (7)	−0.0043 (8)
N8	0.0198 (8)	0.0265 (11)	0.0194 (9)	0.0058 (7)	−0.0020 (7)	0.0033 (8)
N9	0.0359 (10)	0.0323 (11)	0.0196 (9)	0.0114 (8)	−0.0024 (7)	−0.0016 (7)
C53	0.0187 (10)	0.0305 (13)	0.0143 (10)	0.0074 (9)	−0.0009 (8)	−0.0007 (9)
C54	0.0177 (9)	0.0247 (12)	0.0154 (10)	0.0012 (8)	−0.0023 (8)	−0.0041 (9)
C55	0.0177 (9)	0.0241 (12)	0.0183 (10)	0.0066 (8)	0.0007 (8)	0.0002 (8)
C56	0.0213 (10)	0.0232 (12)	0.0201 (10)	0.0073 (9)	0.0007 (8)	0.0016 (8)
C57	0.0266 (10)	0.0276 (12)	0.0224 (10)	0.0120 (9)	0.0013 (8)	0.0004 (8)
C58	0.0266 (10)	0.0324 (13)	0.0199 (10)	0.0141 (9)	0.0043 (8)	0.0040 (8)
C59	0.0289 (10)	0.0279 (12)	0.0197 (10)	0.0150 (9)	−0.0016 (8)	−0.0013 (8)
C60	0.0239 (10)	0.0276 (12)	0.0259 (10)	0.0081 (8)	−0.0003 (8)	0.0003 (8)
C61	0.0268 (10)	0.0274 (11)	0.0213 (10)	0.0097 (9)	0.0023 (8)	0.0026 (8)
C62	0.0414 (13)	0.0406 (15)	0.0234 (11)	0.0123 (11)	−0.0006 (9)	−0.0021 (11)
C63	0.0341 (11)	0.0342 (12)	0.0252 (10)	0.0122 (10)	−0.0070 (8)	−0.0052 (9)
C64	0.0381 (12)	0.0194 (12)	0.0223 (11)	0.0025 (10)	−0.0042 (9)	0.0003 (9)
C65	0.0562 (17)	0.0307 (14)	0.0274 (12)	0.0247 (13)	−0.0144 (11)	−0.0050 (10)
C66	0.0446 (16)	0.059 (2)	0.074 (2)	0.0350 (15)	−0.0091 (15)	−0.0248 (17)
C67	0.0268 (11)	0.0241 (12)	0.0151 (10)	0.0098 (9)	0.0005 (8)	0.0004 (9)
C68	0.0250 (11)	0.0280 (13)	0.0237 (11)	0.0019 (10)	−0.0035 (9)	0.0017 (10)
C69	0.0374 (13)	0.0276 (13)	0.0220 (11)	0.0028 (11)	0.0015 (10)	0.0061 (10)
C70	0.0399 (13)	0.0336 (15)	0.0205 (11)	0.0168 (11)	−0.0031 (10)	−0.0009 (10)
C71	0.0293 (12)	0.0454 (16)	0.0188 (11)	0.0145 (11)	−0.0058 (9)	0.0006 (11)
C72	0.0247 (11)	0.0410 (15)	0.0180 (11)	0.0057 (11)	−0.0003 (9)	0.0017 (10)
C73	0.0171 (9)	0.0217 (12)	0.0254 (11)	0.0052 (8)	−0.0013 (9)	0.0009 (10)
C74	0.0238 (11)	0.0257 (13)	0.0157 (10)	0.0018 (9)	0.0020 (8)	−0.0015 (10)
C75	0.0314 (12)	0.0326 (14)	0.0222 (12)	0.0092 (10)	0.0016 (9)	−0.0067 (10)
C76	0.0302 (12)	0.0224 (13)	0.0359 (14)	0.0051 (10)	−0.0031 (10)	−0.0088 (10)
C77	0.0269 (11)	0.0302 (14)	0.0350 (13)	0.0056 (10)	−0.0066 (10)	0.0042 (11)
C78	0.0222 (10)	0.0309 (13)	0.0201 (11)	0.0071 (9)	−0.0033 (8)	0.0044 (9)
N10	0.0244 (9)	0.0181 (10)	0.0192 (9)	0.0040 (7)	0.0019 (7)	−0.0023 (7)
N11	0.0211 (8)	0.0265 (11)	0.0187 (9)	0.0049 (7)	−0.0019 (7)	0.0018 (8)
N12	0.0454 (11)	0.0376 (12)	0.0196 (9)	0.0154 (9)	−0.0072 (8)	−0.0016 (8)
C79	0.0201 (10)	0.0221 (12)	0.0154 (10)	0.0051 (9)	−0.0015 (8)	−0.0054 (9)
C80	0.0163 (9)	0.0294 (14)	0.0184 (11)	0.0020 (9)	−0.0002 (8)	−0.0023 (10)
C81	0.0208 (10)	0.0299 (13)	0.0164 (10)	0.0076 (9)	0.0017 (8)	−0.0017 (9)
C82	0.0241 (10)	0.0307 (14)	0.0202 (10)	0.0080 (9)	−0.0014 (8)	−0.0017 (9)
C83	0.0308 (11)	0.0273 (12)	0.0213 (10)	0.0086 (9)	−0.0003 (8)	0.0002 (8)
C84	0.0315 (11)	0.0260 (12)	0.0255 (10)	0.0084 (9)	−0.0058 (8)	0.0024 (8)
C85	0.0289 (11)	0.0314 (13)	0.0192 (10)	0.0062 (9)	−0.0024 (8)	0.0002 (9)
C86	0.0413 (13)	0.0289 (13)	0.0208 (10)	0.0152 (10)	−0.0014 (9)	−0.0022 (9)
C87	0.0382 (12)	0.0300 (13)	0.0198 (10)	0.0138 (10)	−0.0013 (8)	0.0022 (8)
C88	0.0421 (13)	0.0365 (14)	0.0233 (11)	0.0097 (11)	−0.0091 (9)	0.0020 (9)
C89	0.0631 (17)	0.0432 (16)	0.0205 (11)	0.0209 (14)	−0.0049 (10)	−0.0017 (10)
C90	0.0384 (12)	0.0193 (12)	0.0208 (11)	0.0062 (10)	−0.0042 (9)	−0.0008 (9)
C91	0.0500 (14)	0.0241 (13)	0.0255 (12)	0.0179 (11)	−0.0108 (10)	−0.0028 (10)
C92	0.0503 (17)	0.0341 (16)	0.079 (2)	0.0255 (13)	0.0181 (15)	0.0077 (14)
C93	0.0282 (11)	0.0188 (12)	0.0174 (11)	0.0089 (9)	0.0014 (8)	−0.0006 (9)
C94	0.0257 (11)	0.0309 (13)	0.0256 (11)	0.0002 (10)	−0.0063 (10)	0.0022 (11)
C95	0.0390 (14)	0.0230 (13)	0.0242 (12)	0.0021 (10)	0.0004 (10)	0.0065 (10)
C96	0.0400 (13)	0.0300 (14)	0.0190 (11)	0.0159 (11)	−0.0041 (10)	−0.0024 (10)
C97	0.0318 (12)	0.0438 (16)	0.0195 (11)	0.0143 (11)	−0.0060 (10)	−0.0004 (11)
C98	0.0259 (12)	0.0409 (15)	0.0194 (11)	0.0066 (11)	0.0025 (9)	0.0014 (10)
C99	0.0163 (9)	0.0184 (12)	0.0202 (10)	0.0047 (8)	−0.0010 (8)	−0.0022 (9)
C100	0.0253 (11)	0.0226 (13)	0.0156 (10)	0.0020 (9)	0.0041 (9)	0.0005 (10)
C101	0.0299 (12)	0.0303 (14)	0.0225 (11)	0.0097 (10)	0.0024 (9)	−0.0072 (10)
C102	0.0277 (11)	0.0214 (12)	0.0262 (11)	0.0057 (9)	0.0019 (9)	−0.0035 (9)
C103	0.0221 (10)	0.0241 (12)	0.0323 (12)	0.0063 (9)	−0.0028 (9)	0.0013 (10)
C104	0.0187 (10)	0.0260 (12)	0.0206 (10)	0.0058 (9)	0.0003 (8)	0.0048 (9)

### Geometric parameters (Å, º)

**Table:**

N1—C1	1.350 (3)	C37—H37A	0.9800
N1—C3	1.401 (3)	C38—H38B	0.9900
N1—C12	1.426 (4)	C38—H38A	0.9900
N2—C2	1.395 (3)	C39—H39	0.9500
N2—C3	1.316 (3)	C40—H40A	0.9500
N3—C7	1.369 (3)	C40—H40B	0.9500
N3—C10	1.457 (3)	C42—H42	0.9500
N3—C11	1.453 (4)	C43—H43	0.9500
N4—C27	1.353 (3)	C44—H44	0.9500
N4—C29	1.395 (3)	C45—H45	0.9500
N4—C38	1.434 (4)	C46—H46	0.9500
N5—C29	1.308 (3)	C48—H48	0.9500
N5—C28	1.396 (3)	C49—H49	0.9500
N6—C33	1.382 (3)	C50—H50	0.9500
N6—C36	1.448 (3)	C51—H51	0.9500
N6—C37	1.449 (3)	C52—H52	0.9500
N7—C53	1.420 (3)	C53—C67	1.480 (3)
N7—C64	1.460 (4)	C53—C54	1.343 (4)
N7—C55	1.348 (3)	C54—C73	1.501 (4)
N8—C55	1.323 (3)	C55—C56	1.477 (3)
N8—C54	1.377 (3)	C56—C57	1.395 (3)
N9—C59	1.383 (3)	C56—C61	1.389 (3)
N9—C62	1.432 (3)	C57—C58	1.386 (3)
N9—C63	1.458 (3)	C58—C59	1.400 (3)
N10—C81	1.346 (3)	C59—C60	1.406 (3)
N10—C90	1.469 (4)	C60—C61	1.388 (3)
N10—C79	1.426 (3)	C64—C65	1.506 (4)
N11—C81	1.326 (3)	C65—C66	1.293 (5)
N11—C80	1.371 (3)	C67—C72	1.401 (4)
N12—C88	1.454 (3)	C67—C68	1.378 (4)
N12—C85	1.377 (3)	C68—C69	1.372 (4)
N12—C89	1.434 (3)	C69—C70	1.380 (4)
C1—C2	1.399 (3)	C70—C71	1.364 (4)
C1—C15	1.481 (3)	C71—C72	1.396 (4)
C2—C21	1.440 (3)	C73—C74	1.380 (3)
C3—C4	1.468 (3)	C73—C78	1.414 (4)
C4—C5	1.404 (3)	C74—C75	1.392 (4)
C4—C9	1.389 (3)	C75—C76	1.351 (4)
C5—C6	1.368 (3)	C76—C77	1.395 (4)
C6—C7	1.405 (3)	C77—C78	1.387 (4)
C7—C8	1.414 (3)	C57—H57	0.9500
C8—C9	1.373 (3)	C58—H58	0.9500
C12—C13	1.423 (4)	C60—H60	0.9500
C13—C14	1.409 (4)	C61—H61	0.9500
C15—C16	1.407 (4)	C62—H62B	0.9800
C15—C20	1.385 (4)	C62—H62C	0.9800
C16—C17	1.393 (4)	C62—H62A	0.9800
C17—C18	1.385 (4)	C63—H63B	0.9800
C18—C19	1.398 (4)	C63—H63C	0.9800
C19—C20	1.376 (4)	C63—H63A	0.9800
C21—C22	1.423 (3)	C64—H64A	0.9900
C21—C26	1.376 (4)	C64—H64B	0.9900
C22—C23	1.373 (4)	C65—H65	0.9500
C23—C24	1.415 (4)	C66—H66A	0.9500
C24—C25	1.383 (4)	C66—H66B	0.9500
C25—C26	1.385 (4)	C68—H68	0.9500
C5—H5	0.9500	C69—H69	0.9500
C6—H6	0.9500	C70—H70	0.9500
C8—H8	0.9500	C71—H71	0.9500
C9—H9	0.9500	C72—H72	0.9500
C10—H10B	0.9800	C74—H74	0.9500
C10—H10A	0.9800	C75—H75	0.9500
C10—H10C	0.9800	C76—H76	0.9500
C11—H11C	0.9800	C77—H77	0.9500
C11—H11B	0.9800	C78—H78	0.9500
C11—H11A	0.9800	C79—C80	1.343 (3)
C12—H12B	0.9900	C79—C93	1.480 (3)
C12—H12A	0.9900	C80—C99	1.496 (3)
C13—H13	0.9500	C81—C82	1.474 (3)
C14—H14B	0.9500	C82—C83	1.395 (3)
C14—H14A	0.9500	C82—C87	1.385 (3)
C16—H16	0.9500	C83—C84	1.384 (3)
C17—H17	0.9500	C84—C85	1.403 (3)
C18—H18	0.9500	C85—C86	1.396 (3)
C19—H19	0.9500	C86—C87	1.385 (3)
C20—H20	0.9500	C90—C91	1.463 (4)
C22—H22	0.9500	C91—C92	1.295 (5)
C23—H23	0.9500	C93—C94	1.380 (4)
C24—H24	0.9500	C93—C98	1.403 (4)
C25—H25	0.9500	C94—C95	1.376 (4)
C26—H26	0.9500	C95—C96	1.378 (4)
C27—C41	1.486 (3)	C96—C97	1.362 (4)
C27—C28	1.401 (4)	C97—C98	1.399 (4)
C28—C47	1.432 (4)	C99—C100	1.379 (3)
C29—C30	1.465 (3)	C99—C104	1.413 (3)
C30—C31	1.392 (3)	C100—C101	1.397 (4)
C30—C35	1.404 (3)	C101—C102	1.352 (4)
C31—C32	1.385 (3)	C102—C103	1.397 (4)
C32—C33	1.403 (3)	C103—C104	1.391 (4)
C33—C34	1.407 (3)	C83—H83	0.9500
C34—C35	1.380 (3)	C84—H84	0.9500
C38—C39	1.453 (6)	C86—H86	0.9500
C39—C40	1.378 (5)	C87—H87	0.9500
C41—C46	1.389 (4)	C88—H88A	0.9800
C41—C42	1.402 (4)	C88—H88B	0.9800
C42—C43	1.396 (3)	C88—H88C	0.9800
C43—C44	1.383 (4)	C89—H89A	0.9800
C44—C45	1.399 (4)	C89—H89B	0.9800
C45—C46	1.372 (4)	C89—H89C	0.9800
C47—C48	1.426 (3)	C90—H90A	0.9900
C47—C52	1.382 (4)	C90—H90B	0.9900
C48—C49	1.367 (4)	C91—H91	0.9500
C49—C50	1.422 (4)	C92—H92A	0.9500
C50—C51	1.378 (4)	C92—H92B	0.9500
C51—C52	1.391 (4)	C94—H94	0.9500
C31—H31	0.9500	C95—H95	0.9500
C32—H32	0.9500	C96—H96	0.9500
C34—H34	0.9500	C97—H97	0.9500
C35—H35	0.9500	C98—H98	0.9500
C36—H36A	0.9800	C100—H100	0.9500
C36—H36B	0.9800	C101—H101	0.9500
C36—H36C	0.9800	C102—H102	0.9500
C37—H37B	0.9800	C103—H103	0.9500
C37—H37C	0.9800	C104—H104	0.9500
			
C1—N1—C3	106.94 (19)	C46—C45—H45	120.00
C1—N1—C12	125.0 (2)	C44—C45—H45	120.00
C3—N1—C12	128.02 (18)	C41—C46—H46	120.00
C2—N2—C3	106.88 (18)	C45—C46—H46	120.00
C7—N3—C10	120.67 (18)	C49—C48—H48	119.00
C7—N3—C11	120.21 (19)	C47—C48—H48	119.00
C10—N3—C11	118.63 (18)	C50—C49—H49	120.00
C27—N4—C38	125.9 (2)	C48—C49—H49	120.00
C29—N4—C38	126.10 (19)	C49—C50—H50	121.00
C27—N4—C29	106.6 (2)	C51—C50—H50	121.00
C28—N5—C29	107.0 (2)	C50—C51—H51	119.00
C36—N6—C37	116.90 (18)	C52—C51—H51	120.00
C33—N6—C37	118.80 (18)	C47—C52—H52	119.00
C33—N6—C36	119.69 (19)	C51—C52—H52	119.00
C53—N7—C55	107.3 (2)	N7—C53—C67	121.4 (2)
C53—N7—C64	125.36 (19)	N7—C53—C54	103.77 (19)
C55—N7—C64	126.43 (19)	C54—C53—C67	134.8 (2)
C54—N8—C55	104.8 (2)	N8—C54—C53	112.5 (2)
C62—N9—C63	116.75 (19)	N8—C54—C73	120.7 (2)
C59—N9—C63	118.84 (18)	C53—C54—C73	126.7 (2)
C59—N9—C62	119.53 (19)	N7—C55—N8	111.7 (2)
C79—N10—C81	107.76 (19)	N7—C55—C56	123.6 (2)
C81—N10—C90	129.2 (2)	N8—C55—C56	124.7 (2)
C79—N10—C90	122.98 (18)	C57—C56—C61	117.76 (19)
C80—N11—C81	105.6 (2)	C55—C56—C57	119.61 (18)
C88—N12—C89	118.82 (18)	C55—C56—C61	122.63 (19)
C85—N12—C89	119.71 (19)	C56—C57—C58	121.48 (18)
C85—N12—C88	120.90 (19)	C57—C58—C59	120.79 (17)
N1—C1—C2	107.4 (2)	C58—C59—C60	117.75 (16)
N1—C1—C15	123.9 (2)	N9—C59—C58	121.66 (17)
C2—C1—C15	128.7 (2)	N9—C59—C60	120.58 (18)
N2—C2—C21	120.82 (19)	C59—C60—C61	120.66 (18)
C1—C2—C21	131.1 (2)	C56—C61—C60	121.55 (18)
N2—C2—C1	108.02 (18)	N7—C64—C65	112.6 (2)
N2—C3—C4	123.32 (19)	C64—C65—C66	126.7 (3)
N1—C3—N2	110.71 (19)	C53—C67—C68	121.3 (3)
N1—C3—C4	125.96 (18)	C53—C67—C72	119.5 (2)
C3—C4—C5	117.81 (18)	C68—C67—C72	119.3 (2)
C5—C4—C9	116.6 (2)	C67—C68—C69	120.2 (3)
C3—C4—C9	125.53 (19)	C68—C69—C70	121.2 (3)
C4—C5—C6	122.09 (18)	C69—C70—C71	119.2 (3)
C5—C6—C7	121.31 (19)	C70—C71—C72	120.9 (3)
N3—C7—C8	120.84 (19)	C67—C72—C71	119.2 (3)
C6—C7—C8	116.6 (2)	C54—C73—C78	119.6 (2)
N3—C7—C6	122.52 (19)	C54—C73—C74	120.8 (2)
C7—C8—C9	121.1 (2)	C74—C73—C78	119.7 (2)
C4—C9—C8	122.2 (2)	C73—C74—C75	119.2 (3)
N1—C12—C13	116.9 (2)	C74—C75—C76	122.1 (2)
C12—C13—C14	125.0 (3)	C75—C76—C77	119.5 (3)
C16—C15—C20	118.3 (2)	C76—C77—C78	120.3 (3)
C1—C15—C16	120.4 (2)	C73—C78—C77	119.3 (2)
C1—C15—C20	121.2 (2)	C56—C57—H57	119.00
C15—C16—C17	120.5 (3)	C58—C57—H57	119.00
C16—C17—C18	120.3 (3)	C57—C58—H58	120.00
C17—C18—C19	119.1 (2)	C59—C58—H58	120.00
C18—C19—C20	120.6 (3)	C59—C60—H60	120.00
C15—C20—C19	121.3 (3)	C61—C60—H60	120.00
C22—C21—C26	117.1 (2)	C56—C61—H61	119.00
C2—C21—C22	120.9 (2)	C60—C61—H61	119.00
C2—C21—C26	122.0 (2)	H62A—C62—H62B	109.00
C21—C22—C23	121.6 (3)	N9—C62—H62A	110.00
C22—C23—C24	119.9 (2)	N9—C62—H62B	110.00
C23—C24—C25	118.6 (3)	N9—C62—H62C	110.00
C24—C25—C26	120.7 (3)	H62A—C62—H62C	109.00
C21—C26—C25	122.1 (2)	H62B—C62—H62C	109.00
C4—C5—H5	119.00	N9—C63—H63A	109.00
C6—C5—H5	119.00	N9—C63—H63B	110.00
C7—C6—H6	119.00	N9—C63—H63C	110.00
C5—C6—H6	119.00	H63A—C63—H63B	109.00
C9—C8—H8	119.00	H63A—C63—H63C	110.00
C7—C8—H8	119.00	H63B—C63—H63C	109.00
C4—C9—H9	119.00	H64A—C64—H64B	108.00
C8—C9—H9	119.00	N7—C64—H64B	109.00
H10B—C10—H10C	109.00	N7—C64—H64A	109.00
H10A—C10—H10C	109.00	C65—C64—H64A	109.00
N3—C10—H10C	110.00	C65—C64—H64B	109.00
H10A—C10—H10B	109.00	C64—C65—H65	117.00
N3—C10—H10A	110.00	C66—C65—H65	117.00
N3—C10—H10B	109.00	C65—C66—H66A	120.00
N3—C11—H11B	110.00	C65—C66—H66B	120.00
N3—C11—H11C	110.00	H66A—C66—H66B	120.00
N3—C11—H11A	109.00	C67—C68—H68	120.00
H11B—C11—H11C	109.00	C69—C68—H68	120.00
H11A—C11—H11B	109.00	C68—C69—H69	119.00
H11A—C11—H11C	109.00	C70—C69—H69	119.00
N1—C12—H12A	108.00	C69—C70—H70	120.00
C13—C12—H12A	108.00	C71—C70—H70	120.00
H12A—C12—H12B	107.00	C72—C71—H71	120.00
N1—C12—H12B	108.00	C70—C71—H71	119.00
C13—C12—H12B	108.00	C67—C72—H72	120.00
C12—C13—H13	118.00	C71—C72—H72	120.00
C14—C13—H13	117.00	C75—C74—H74	120.00
C13—C14—H14A	120.00	C73—C74—H74	120.00
H14A—C14—H14B	120.00	C74—C75—H75	119.00
C13—C14—H14B	120.00	C76—C75—H75	119.00
C17—C16—H16	120.00	C75—C76—H76	120.00
C15—C16—H16	120.00	C77—C76—H76	120.00
C16—C17—H17	120.00	C76—C77—H77	120.00
C18—C17—H17	120.00	C78—C77—H77	120.00
C19—C18—H18	121.00	C73—C78—H78	120.00
C17—C18—H18	120.00	C77—C78—H78	120.00
C18—C19—H19	120.00	N10—C79—C80	103.42 (19)
C20—C19—H19	120.00	N10—C79—C93	122.61 (19)
C19—C20—H20	119.00	C80—C79—C93	134.0 (2)
C15—C20—H20	119.00	N11—C80—C79	112.4 (2)
C21—C22—H22	119.00	N11—C80—C99	120.5 (2)
C23—C22—H22	119.00	C79—C80—C99	127.1 (2)
C24—C23—H23	120.00	N10—C81—N11	110.79 (19)
C22—C23—H23	120.00	N10—C81—C82	126.7 (2)
C25—C24—H24	121.00	N11—C81—C82	122.5 (2)
C23—C24—H24	121.00	C81—C82—C83	118.6 (2)
C24—C25—H25	120.00	C81—C82—C87	124.0 (2)
C26—C25—H25	120.00	C83—C82—C87	117.17 (19)
C21—C26—H26	119.00	C82—C83—C84	121.75 (19)
C25—C26—H26	119.00	C83—C84—C85	120.99 (19)
N4—C27—C28	107.5 (2)	N12—C85—C84	121.65 (19)
C28—C27—C41	129.4 (2)	N12—C85—C86	121.4 (2)
N4—C27—C41	123.1 (2)	C84—C85—C86	117.0 (2)
N5—C28—C47	121.6 (2)	C85—C86—C87	121.5 (2)
N5—C28—C27	107.6 (2)	C82—C87—C86	121.6 (2)
C27—C28—C47	130.8 (2)	N10—C90—C91	114.3 (2)
N4—C29—N5	111.2 (2)	C90—C91—C92	127.7 (3)
N5—C29—C30	126.5 (2)	C79—C93—C94	122.0 (2)
N4—C29—C30	122.4 (2)	C79—C93—C98	119.4 (2)
C29—C30—C31	120.1 (2)	C94—C93—C98	118.6 (2)
C31—C30—C35	117.68 (19)	C93—C94—C95	120.8 (3)
C29—C30—C35	122.2 (2)	C94—C95—C96	120.7 (3)
C30—C31—C32	121.51 (19)	C95—C96—C97	119.5 (2)
C31—C32—C33	121.02 (17)	C96—C97—C98	120.8 (3)
N6—C33—C34	121.07 (18)	C93—C98—C97	119.5 (3)
N6—C33—C32	121.52 (17)	C80—C99—C100	121.1 (2)
C32—C33—C34	117.40 (16)	C80—C99—C104	119.3 (2)
C33—C34—C35	121.21 (18)	C100—C99—C104	119.6 (2)
C30—C35—C34	121.18 (18)	C99—C100—C101	119.4 (2)
N4—C38—C39	115.3 (2)	C100—C101—C102	121.7 (2)
C38—C39—C40	126.0 (3)	C101—C102—C103	119.9 (2)
C42—C41—C46	118.8 (2)	C102—C103—C104	119.9 (3)
C27—C41—C42	119.8 (2)	C99—C104—C103	119.6 (2)
C27—C41—C46	121.4 (2)	C82—C83—H83	119.00
C41—C42—C43	120.3 (3)	C84—C83—H83	119.00
C42—C43—C44	120.1 (3)	C83—C84—H84	119.00
C43—C44—C45	119.4 (2)	C85—C84—H84	120.00
C44—C45—C46	120.6 (3)	C85—C86—H86	119.00
C41—C46—C45	120.9 (3)	C87—C86—H86	119.00
C28—C47—C48	121.3 (2)	C82—C87—H87	119.00
C48—C47—C52	116.8 (2)	C86—C87—H87	119.00
C28—C47—C52	122.0 (2)	N12—C88—H88A	109.00
C47—C48—C49	122.2 (3)	N12—C88—H88B	109.00
C48—C49—C50	119.6 (2)	N12—C88—H88C	109.00
C49—C50—C51	118.6 (3)	H88A—C88—H88B	110.00
C50—C51—C52	121.1 (3)	H88A—C88—H88C	109.00
C47—C52—C51	121.7 (2)	H88B—C88—H88C	109.00
C32—C31—H31	119.00	N12—C89—H89A	110.00
C30—C31—H31	119.00	N12—C89—H89B	109.00
C31—C32—H32	120.00	N12—C89—H89C	110.00
C33—C32—H32	119.00	H89A—C89—H89B	109.00
C33—C34—H34	119.00	H89A—C89—H89C	110.00
C35—C34—H34	119.00	H89B—C89—H89C	109.00
C30—C35—H35	119.00	N10—C90—H90A	109.00
C34—C35—H35	119.00	N10—C90—H90B	109.00
H36A—C36—H36C	110.00	C91—C90—H90A	109.00
H36B—C36—H36C	109.00	C91—C90—H90B	109.00
H36A—C36—H36B	109.00	H90A—C90—H90B	108.00
N6—C36—H36C	109.00	C90—C91—H91	116.00
N6—C36—H36B	110.00	C92—C91—H91	116.00
N6—C36—H36A	109.00	C91—C92—H92A	120.00
N6—C37—H37C	109.00	C91—C92—H92B	120.00
N6—C37—H37B	109.00	H92A—C92—H92B	120.00
H37B—C37—H37C	110.00	C93—C94—H94	120.00
N6—C37—H37A	109.00	C95—C94—H94	120.00
H37A—C37—H37C	109.00	C94—C95—H95	120.00
H37A—C37—H37B	109.00	C96—C95—H95	120.00
C39—C38—H38A	108.00	C95—C96—H96	120.00
C39—C38—H38B	108.00	C97—C96—H96	120.00
H38A—C38—H38B	107.00	C96—C97—H97	120.00
N4—C38—H38B	109.00	C98—C97—H97	120.00
N4—C38—H38A	108.00	C93—C98—H98	120.00
C40—C39—H39	117.00	C97—C98—H98	120.00
C38—C39—H39	117.00	C99—C100—H100	120.00
H40A—C40—H40B	120.00	C101—C100—H100	120.00
C39—C40—H40B	120.00	C100—C101—H101	119.00
C39—C40—H40A	120.00	C102—C101—H101	119.00
C41—C42—H42	120.00	C101—C102—H102	120.00
C43—C42—H42	120.00	C103—C102—H102	120.00
C42—C43—H43	120.00	C102—C103—H103	120.00
C44—C43—H43	120.00	C104—C103—H103	120.00
C43—C44—H44	120.00	C99—C104—H104	120.00
C45—C44—H44	120.00	C103—C104—H104	120.00
			
C3—N1—C1—C2	1.3 (2)	N4—C27—C41—C46	115.0 (3)
C12—N1—C1—C2	−175.8 (2)	N5—C28—C47—C52	−27.2 (4)
C3—N1—C1—C15	−176.8 (2)	C27—C28—C47—C48	−25.5 (4)
C12—N1—C1—C15	6.1 (4)	N5—C28—C47—C48	151.4 (3)
C12—N1—C3—N2	176.3 (2)	C27—C28—C47—C52	155.9 (3)
C1—N1—C3—C4	178.2 (2)	N4—C29—C30—C31	−123.9 (3)
C12—N1—C3—C4	−4.8 (4)	N5—C29—C30—C31	54.5 (4)
C1—N1—C12—C13	−79.2 (3)	N4—C29—C30—C35	58.7 (4)
C3—N1—C12—C13	104.3 (3)	N5—C29—C30—C35	−122.8 (3)
C1—N1—C3—N2	−0.7 (2)	C31—C30—C35—C34	−0.6 (3)
C2—N2—C3—N1	−0.2 (2)	C29—C30—C31—C32	−177.1 (2)
C3—N2—C2—C21	−176.94 (19)	C35—C30—C31—C32	0.4 (3)
C2—N2—C3—C4	−179.15 (19)	C29—C30—C35—C34	176.8 (2)
C3—N2—C2—C1	1.0 (2)	C30—C31—C32—C33	0.2 (3)
C10—N3—C7—C6	5.2 (3)	C31—C32—C33—N6	−179.73 (19)
C11—N3—C7—C8	−2.9 (3)	C31—C32—C33—C34	−0.6 (3)
C11—N3—C7—C6	177.0 (2)	C32—C33—C34—C35	0.4 (3)
C10—N3—C7—C8	−174.8 (2)	N6—C33—C34—C35	179.53 (19)
C29—N4—C38—C39	86.9 (3)	C33—C34—C35—C30	0.2 (3)
C27—N4—C29—N5	−1.3 (3)	N4—C38—C39—C40	0.4 (4)
C38—N4—C29—N5	−168.4 (2)	C46—C41—C42—C43	−2.0 (4)
C27—N4—C38—C39	−77.9 (3)	C42—C41—C46—C45	2.2 (4)
C29—N4—C27—C28	2.1 (3)	C27—C41—C46—C45	179.7 (3)
C38—N4—C27—C41	−10.1 (4)	C27—C41—C42—C43	−179.5 (3)
C38—N4—C27—C28	169.3 (2)	C41—C42—C43—C44	1.6 (4)
C29—N4—C27—C41	−177.2 (2)	C42—C43—C44—C45	−1.4 (4)
C38—N4—C29—C30	10.3 (4)	C43—C44—C45—C46	1.6 (4)
C27—N4—C29—C30	177.4 (2)	C44—C45—C46—C41	−2.1 (4)
C29—N5—C28—C47	−176.1 (2)	C48—C47—C52—C51	2.9 (4)
C28—N5—C29—C30	−178.7 (3)	C52—C47—C48—C49	−2.5 (4)
C28—N5—C29—N4	−0.1 (3)	C28—C47—C52—C51	−178.5 (3)
C29—N5—C28—C27	1.4 (3)	C28—C47—C48—C49	178.8 (3)
C37—N6—C33—C34	12.6 (3)	C47—C48—C49—C50	0.8 (5)
C36—N6—C33—C32	−13.1 (3)	C48—C49—C50—C51	0.6 (4)
C37—N6—C33—C32	−168.29 (18)	C49—C50—C51—C52	−0.2 (4)
C36—N6—C33—C34	167.8 (2)	C50—C51—C52—C47	−1.6 (5)
C53—N7—C55—N8	0.9 (3)	N7—C53—C67—C72	−115.6 (3)
C64—N7—C53—C54	−170.8 (2)	C54—C53—C67—C68	−115.7 (3)
C53—N7—C64—C65	79.6 (3)	N7—C53—C67—C68	65.5 (3)
C64—N7—C55—N8	170.2 (2)	C67—C53—C54—C73	6.9 (4)
C55—N7—C53—C67	177.8 (2)	C67—C53—C54—N8	−177.5 (3)
C64—N7—C55—C56	−9.4 (4)	C54—C53—C67—C72	63.3 (4)
C55—N7—C64—C65	−87.9 (3)	N7—C53—C54—N8	1.4 (2)
C55—N7—C53—C54	−1.4 (2)	N7—C53—C54—C73	−174.2 (2)
C53—N7—C55—C56	−178.68 (19)	C53—C54—C73—C74	24.4 (3)
C64—N7—C53—C67	8.3 (3)	N8—C54—C73—C78	29.5 (3)
C55—N8—C54—C73	174.9 (2)	C53—C54—C73—C78	−155.3 (2)
C54—N8—C55—C56	179.6 (2)	N8—C54—C73—C74	−150.8 (2)
C55—N8—C54—C53	−0.9 (3)	N7—C55—C56—C57	122.8 (2)
C54—N8—C55—N7	0.0 (2)	N8—C55—C56—C61	122.4 (2)
C62—N9—C59—C60	−167.6 (2)	N7—C55—C56—C61	−58.1 (3)
C62—N9—C59—C58	13.8 (3)	N8—C55—C56—C57	−56.7 (3)
C63—N9—C59—C58	168.25 (18)	C55—C56—C57—C58	178.8 (2)
C63—N9—C59—C60	−13.1 (3)	C55—C56—C61—C60	−178.4 (2)
C81—N10—C90—C91	−102.2 (3)	C61—C56—C57—C58	−0.4 (3)
C79—N10—C81—C82	−179.8 (2)	C57—C56—C61—C60	0.7 (3)
C81—N10—C79—C93	178.9 (2)	C56—C57—C58—C59	−0.6 (3)
C90—N10—C79—C80	175.5 (2)	C57—C58—C59—C60	1.1 (3)
C79—N10—C90—C91	81.9 (3)	C57—C58—C59—N9	179.77 (19)
C90—N10—C81—C82	3.8 (4)	C58—C59—C60—C61	−0.7 (3)
C90—N10—C79—C93	−4.4 (3)	N9—C59—C60—C61	−179.43 (19)
C90—N10—C81—N11	−175.6 (2)	C59—C60—C61—C56	−0.2 (3)
C79—N10—C81—N11	0.8 (2)	N7—C64—C65—C66	−9.6 (4)
C81—N10—C79—C80	−1.2 (2)	C53—C67—C68—C69	176.0 (3)
C80—N11—C81—C82	−179.5 (2)	C53—C67—C72—C71	−175.3 (3)
C80—N11—C81—N10	−0.1 (2)	C68—C67—C72—C71	3.7 (4)
C81—N11—C80—C79	−0.7 (3)	C72—C67—C68—C69	−3.0 (4)
C81—N11—C80—C99	177.25 (19)	C67—C68—C69—C70	1.9 (4)
C89—N12—C85—C84	−176.4 (2)	C68—C69—C70—C71	−1.5 (4)
C88—N12—C85—C84	−5.2 (3)	C69—C70—C71—C72	2.3 (4)
C89—N12—C85—C86	3.1 (3)	C70—C71—C72—C67	−3.4 (4)
C88—N12—C85—C86	174.3 (2)	C74—C73—C78—C77	−1.1 (3)
N1—C1—C2—C21	176.2 (2)	C54—C73—C78—C77	178.7 (2)
N1—C1—C15—C16	−71.0 (3)	C54—C73—C74—C75	−178.6 (2)
C2—C1—C15—C20	−64.1 (4)	C78—C73—C74—C75	1.2 (3)
C15—C1—C2—N2	176.6 (2)	C73—C74—C75—C76	−0.9 (4)
C15—C1—C2—C21	−5.8 (4)	C74—C75—C76—C77	0.5 (4)
N1—C1—C15—C20	113.6 (3)	C75—C76—C77—C78	−0.3 (4)
C2—C1—C15—C16	111.3 (3)	C76—C77—C78—C73	0.6 (4)
N1—C1—C2—N2	−1.4 (2)	C80—C79—C93—C94	−109.7 (3)
C1—C2—C21—C26	154.8 (3)	N10—C79—C93—C94	70.2 (3)
N2—C2—C21—C26	−27.8 (3)	C93—C79—C80—C99	3.3 (4)
N2—C2—C21—C22	151.5 (2)	N10—C79—C93—C98	−111.8 (3)
C1—C2—C21—C22	−25.9 (4)	N10—C79—C80—C99	−176.6 (2)
N1—C3—C4—C5	154.8 (2)	C93—C79—C80—N11	−178.9 (2)
N2—C3—C4—C5	−26.5 (3)	C80—C79—C93—C98	68.3 (4)
N2—C3—C4—C9	150.8 (2)	N10—C79—C80—N11	1.2 (2)
N1—C3—C4—C9	−28.0 (3)	N11—C80—C99—C104	28.1 (3)
C5—C4—C9—C8	0.5 (3)	C79—C80—C99—C104	−154.3 (2)
C3—C4—C5—C6	176.21 (19)	N11—C80—C99—C100	−152.0 (2)
C3—C4—C9—C8	−176.8 (2)	C79—C80—C99—C100	25.7 (3)
C9—C4—C5—C6	−1.3 (3)	N10—C81—C82—C87	30.9 (4)
C4—C5—C6—C7	1.0 (3)	N10—C81—C82—C83	−155.6 (2)
C5—C6—C7—C8	0.1 (3)	N11—C81—C82—C83	23.8 (3)
C5—C6—C7—N3	−179.8 (2)	N11—C81—C82—C87	−149.8 (2)
N3—C7—C8—C9	179.0 (2)	C81—C82—C87—C86	175.8 (2)
C6—C7—C8—C9	−1.0 (3)	C83—C82—C87—C86	2.2 (3)
C7—C8—C9—C4	0.6 (4)	C87—C82—C83—C84	−1.3 (3)
N1—C12—C13—C14	−8.5 (3)	C81—C82—C83—C84	−175.3 (2)
C1—C15—C16—C17	−177.7 (3)	C82—C83—C84—C85	−0.2 (3)
C16—C15—C20—C19	2.2 (4)	C83—C84—C85—N12	−179.70 (19)
C1—C15—C20—C19	177.7 (3)	C83—C84—C85—C86	0.8 (3)
C20—C15—C16—C17	−2.2 (4)	N12—C85—C86—C87	−179.4 (2)
C15—C16—C17—C18	2.1 (4)	C84—C85—C86—C87	0.1 (4)
C16—C17—C18—C19	−1.9 (4)	C85—C86—C87—C82	−1.7 (4)
C17—C18—C19—C20	1.9 (4)	N10—C90—C91—C92	−1.3 (4)
C18—C19—C20—C15	−2.1 (4)	C79—C93—C94—C95	175.1 (3)
C2—C21—C26—C25	−178.1 (2)	C98—C93—C94—C95	−2.9 (4)
C2—C21—C22—C23	178.4 (2)	C79—C93—C98—C97	−174.5 (2)
C26—C21—C22—C23	−2.2 (4)	C94—C93—C98—C97	3.5 (4)
C22—C21—C26—C25	2.6 (4)	C93—C94—C95—C96	1.7 (4)
C21—C22—C23—C24	0.7 (4)	C94—C95—C96—C97	−1.2 (4)
C22—C23—C24—C25	0.6 (4)	C95—C96—C97—C98	1.9 (4)
C23—C24—C25—C26	−0.2 (4)	C96—C97—C98—C93	−3.1 (4)
C24—C25—C26—C21	−1.4 (5)	C80—C99—C100—C101	−178.3 (2)
N4—C27—C28—C47	175.0 (2)	C104—C99—C100—C101	1.7 (3)
C41—C27—C28—N5	177.1 (2)	C80—C99—C104—C103	178.7 (2)
N4—C27—C41—C42	−67.6 (3)	C100—C99—C104—C103	−1.2 (3)
C41—C27—C28—C47	−5.7 (4)	C99—C100—C101—C102	−0.8 (4)
C28—C27—C41—C46	−64.2 (4)	C100—C101—C102—C103	−0.7 (4)
C28—C27—C41—C42	113.2 (3)	C101—C102—C103—C104	1.1 (4)
N4—C27—C28—N5	−2.2 (2)	C102—C103—C104—C99	−0.2 (4)

### Hydrogen-bond geometry (Å, º)

Cg2, Cg8, Cg6, Cg4, Cg10, Cg16, Cg14 and Cg12 are the centroids of the C4–C9, C47–C52, C30–C35, C21–C26, C56–C61, C99–C104, C82–C87 and C73–C78 rings, respectively.

**Table:**

*D*—H···*A*	*D*—H	H···*A*	*D*···*A*	*D*—H···*A*
C66—H66*A*···N7	0.95	2.54	2.864 (4)	100
C92—H92*A*···N10	0.95	2.56	2.876 (4)	100
C10—H10*A*···*Cg*2^i^	0.98	2.71	3.514 (3)	139
C16—H16···*Cg*8	0.95	2.47	3.328 (3)	151
C37—H37*A*···*Cg*6^ii^	0.98	2.77	3.600 (2)	142
C42—H42···*Cg*4^iii^	0.95	2.49	3.345 (3)	150
C63—H63*A*···*Cg*10^iv^	0.98	2.73	3.595 (2)	147
C68—H68···*Cg*16	0.95	2.59	3.390 (3)	142
C88—H88*C*···*Cg*14^v^	0.98	2.74	3.509 (3)	136
C94—H94···*Cg*12^iii^	0.95	2.48	3.340 (3)	150

Symmetry codes: (i) −*x*+1, −*y*, −*z*; (ii) −*x*+2, −*y*+1, −*z*; (iii) *x*+1, *y*+1, *z*; (iv) −*x*+1, −*y*+1, −*z*+1; (v) −*x*+2, −*y*+2, −*z*+1.
